# POST-OSTEOSYNTHESIS INFECTION: EVALUATION OF A HISTORICAL COHORT AND DEVELOPMENT OF A CARE PROTOCOL

**DOI:** 10.1590/1413-785220253304e277810

**Published:** 2025-09-08

**Authors:** Jair Moreira Dias, Pedro Henrique Ananias da Cunha, Marcos Paulo Paro, Mariana Machado Pinheiro, Ibsen Barguine Junqueira Passos, Adriano Fernando Mendes

**Affiliations:** 1 Universidade Federal de Juiz de Fora, Servico de Ortopedia do Hospital Universitario, Juiz de Fora, Minas Gerais, MG, Brazil.

**Keywords:** Trauma, Fracture Fixation, Osteosynthesis, Fracture, Infections, Surgical Wound Infection, Trauma, Fixação de Fratura, Osteossíntese, Infecção, Infecção de Ferida Operatória

## Abstract

**Objective:**

To evaluate patients with post-osteosynthesis infection (POI) in the appendicular skeleton, in a tertiary hospital, and to develop a care protocol for case management, based on cohort data and agreement between attending physicians.

**Method:**

primary, observational, retrospective and comparative study, evaluating cases of POI, from 2014 to 2019, assisted by the service. The outcomes analyzed were length of stay, readmission, fracture site, etiological agent, antibiotic therapy, number of surgeries and clinical outcome after one year (cure or failure, these considered recurrence, amputation or death). Based on this data, a protocol for diagnosing and treating POI was proposed to the institution's orthopedists, with responses assessed in terms of the content validity coefficient, in order to grade compliance with the protocol.

**Results:**

sample of 27 participants, mostly male, with infection in the lower limbs (77.8%), who underwent an average of two surgeries. The average length of stay was 48 days. The median duration of antibiotic therapy was 34 days. Patients with cure criteria used a greater amount of antibiotics compared to other cases (p<0.05). The responses of orthopedists to the management protocol for these cases reached a concordance of 0.91.

**Conclusion:**

The profile of patients and therapy to POI was similar to the literature and supported the development of the care protocol, with high agreement among developers. ***Level of Evidence III; Observational, Retrospective and Comparative Study.***

## RESUMO

**Objetivo:**

Avaliar pacientes com infecção pós osteossíntese (IPO) no esqueleto apendicular, em um hospital terciário, e desenvolver um protocolo assistencial para manejo dos casos, baseado nos dados da coorte e na concordância entre médicos assistentes.

**Método:**

estudo primário, observacional, retrospectivo e comparativo, avaliando casos de IPO de 2014 a 2019, atendidos pelo serviço. Os desfechos analisados foram tempo de internação, local da fratura, agente etiológico, antibioticoterapia, número de cirurgias e desfecho clínico após um ano (cura ou falha, estes considerados recorrência, amputação ou óbito. Baseado nestes dados foi proposto aos ortopedistas da instituição um protocolo de diagnóstico e terapêutica de IPO, com respostas avaliadas quanto ao coeficiente de validade de conteúdo, para graduação da concordância ao protocolo.

**Resultados:**

amostra de 27 participantes, maioria do sexo masculino, com infecção nos membros inferiores (77,8%), submetidos em média a duas cirurgias. O tempo médio de internação foi 48 dias. A mediana do tempo antibioticoterapia foi de 34 dias. Pacientes com critérios de cura utilizaram maior quantidade de antibióticos se comparado aos demais casos (p<0,05). As respostas dos ortopedistas para o protocolo de manejo destes casos obtiveram concordância de 0,91.

**Conclusão:**

O perfil dos enfermos e da terapêutica à IPO foi semelhante à literatura e respaldou o desenvolvimento do protocolo assistencial, com alta concordância entre os desenvolvedores. ***Nível de Evidência III; Estudo observacional, Retrospectivo e Comparativo.***

## INTRODUCTION

Infection related to fractures is a serious complication in the management of skeletal muscle trauma, with impact on quality of life and significant socioeconomic consequences.^1^ Post-osteosynthesis infection (POI) occurs in up to 5% after fixation of low-energy closed fractures and more than 50% after surgical treatment of exposed fractures.^2,3^ The estimated cost is 6.5 times higher than for a patient without infection.^4^ Furthermore, exposes the patient to prolonged hospitalization, the need for multiple surgeries and can result in adverse outcomes, such as recurrence of infection, amputation and even death.^5^

There is little agreement on the best diagnostic and treatment criteria for POI, and discussing them is challenging for surgeons, microbiologists and infectologists.^5^ A systematic review^1^ of 3,711 POI cases, observed that nine different classifications have been described and multiple stages of treatment, most without standardization.^6^ Therefore, it is necessary to evaluate the characteristics of the treatment of patients with POI and build an instrument for standardizing the diagnosis and conduct of these cases. The aim of this study is to identify and characterize patients diagnosed with POI of appendicular skeletal fractures in a tertiary hospital and develop a treatment protocol for this condition based on cohort data and agreement between the assisting physicians.

## MATERIALS AND METHODS

This is a primary, observational, retrospective study of cases of post-osteosynthesis infection (POI), in a university hospital, tertiary level, and that was approved by the CEP institutional. All participants signed the Free and Informed Consent Clause – approval number of the ethics committee protocol CAAE 36755020.0.0000.5133.

The records were accessed on a digital platform and researched the records of adults admitted between January 1, 2014 and December 31, 2019, who underwent surgical treatment of fractures, with a minimum time of admission of seven days and whose codes for admission or identification corresponded to those of identified appendicular fractures or fractures of unspecified level of the upper and/or lower limb. Children under 18 years of age, those with spinal or pelvic fractures, those undergoing elective surgeries, cases of pathological fractures, those diagnosed with tumor diseases and or with prolonged hospitalization time due to clinical incidents not related to POI were excluded. The following, from this sample, evaluated as eligible participants those with diagnostic POI criteria according to Metsemakers^6^ (see below).

From the sample data were cataloged such as gender, age, site of fractures, affected bone region (exclusive diaphyseal or non-exclusive diaphyseal), site of infection, number of surgeries performed, time of hospitalization, readmission, antibiotic used, total time of antibiotic therapy, etiological agents of infection, follow-up time and clinical outcome in the follow-up of at least one year. POI was categorized according to the time of onset of symptoms as acute (less than four weeks), or chronic.^7-9^

The age was divided into less than 30 years classified as young, between 30 and 59 years as adults and older than 60 years as elderly. Regarding the follow-up of fractured appendicular skeleton, they were divided into two groups: upper or lower limbs. Polyfractured individuals were defined as those presenting with more than one fracture site in the appendicular skeleton, regardless of concomitant injuries such as Traumatic brain injury (TBI) abdominal trauma, or spinal cord injury (SCI).

For the diagnosis of POI, the criteria of Metsemakers et al were used,^6^ being confirmatory criteria: 1. Fistula; 2. Collection or rupture of the soft parts with exposure of the focus of the fracture or implant (medium communication with bone or implant); 3. Purulent drainage of the wound or the presence of pus during surgery; 4. Phenotypically similar pathogens, identified by culture from at least two separate bone/deep tissue fragments/implants (including sonication liquid) collected during an operational intervention or at least three tissue samples.

As suggestive POI criteria: clinical - pain (no weight support, increasing over time, new symptom), local redness, edema, temperature rise, fever (single temperature measurement of 38.3 C);^6^ image - radiological signs of bone lyse (at the site of the fracture, around the implant), implant release, bone sequestration (occurring over time), failure in the progression of bone healing (not joining) and presence of periosteal bone formation (e.g., at different locations from the site of the fracture);^6^ laboratory: positive culture (identification of a pathogenic organism from a tissue sample/implant, including sonication fluid evaluation); three laboratory markers, hemostasis sedimentation speed (HSS), white blood cell count (WBC) and reactive CPC.

The cure of the case was considered a good result, being defined as the resolution of the clinical signs of infection; a patient who did not evolve to amputation or death from this etiology. Already unfavorable result, one with recurrence of infection (fistulas, repeated abscesses, or radiographic sign of release of the implant), need for amputation of the affected limb or death due to the infectious cause. Rehabilitation for POI treatment was evaluated as a secondary outcome not related to cure or recurrence, as many cases received stagnant treatment in non-continuous scheduled interventions.

The data on soft parts or the degree of exposure of exposed fractures were not evaluated, as patients were referred to the reference service after primary care in another institution, where such a description was not routinely carried out by the care body. The cohort data were used in drafting a protocol for diagnostic and therapeutic POI (Annex I), proposed to nine assistant orthopedic doctors, which was answered in the period from February 2022 to March 2022. The agreement between the responses was evaluated and determined that if the agreement to the questionnaire obtained a value greater than 80%,^10^ these criteria would be established as POI assistance protocol in the institution.

For descriptive statistics it was performed using the average and the standard deviation (minimum – maximum) for the continuous variables and proportions for the categorial variables. The variables hospital time and antibiotic use time were dichotomized based on the median value. The Qui-Quadrant test was used to test the association between qualitative variables, while the Pearson correlation test was used for quantitative variables. Student's t test was used to test differences between averages. The assumptions of normality and equality of variance were evaluated by the Komolgorov-Smirnov test and Levene test, respectively. The size of the effect (SE) was evaluated by Cohen's *d* and Cramer's V, adopting the classification proposed by (Cohen, 1992),^10^ which is a measure of the magnitude of the difference between two averages, while Cramer's V evaluates the strength of the association between nominal variables. Cohen established criteria for interpreting these values as small, medium or large. The analyses were processed using SPSS software (IBM SPSS Statistics, version 22.0; IBM Corporation). The content validity coefficient (CVC), which is verified by the scale from 0 to 1, the following classification: less than 0.80, is considered unacceptable; from 0.80 to 0.90 as acceptable; above 0.90 as excellent the validity of content, proposed by Hernandez-Nieto (2002)^11^ was used to evaluate the relevance of each item of the instrument and the instrument as a whole. The evaluators used a scale of 0 to 2 points to evaluate the level of relevance, being 0 = Disagrees, 1 = Agrees with reservations and 2 = Agrees. Items should have CVC ≥ 0.80.

## RESULTS

**Figure 1:**
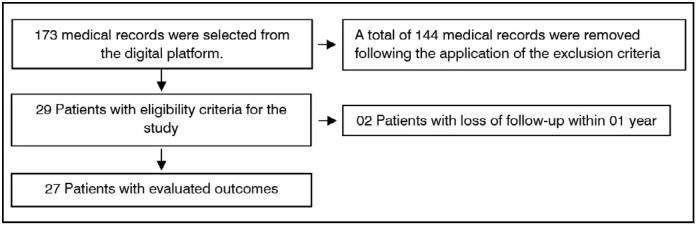
Summary of patient selection and follow-up.

Initial data were collected from 173 patients with fractures, of which those who presented a description of POI treatment and application of exclusion criteria totalized a sample of 27 patients. (Figure 1)

Participants were predominantly male, with an average age of 50 years. Most fractures (55.5%) occurred in the lower limbs. Infection sites were: femur (33.3%), tibia (33.3%), ankle/foot (11.1%), forearm/hand (11.1%), homer (7.4%) and radius (3.7%). The average hospitalization time was 48 days and each patient performed an average of two surgeries. Seventy percent of patients were readmitted, and approximately 93% had positive culture results. (Table 1)

**Table 1:** Sample characteristics (n = 27).

Variables	Average ± SD n	Minimum – Maximum %
Age (years)	49.7 ± 18.7	22.0 – 88.0
**Sex**		
Female	8	29.6%
Male	19	70.4%
Internship (days)	48.2 ± 27.6	17.0 – 123.0
Readmission (yes)	20	70.4%
**fractured bone**		
Arm/hand	3	11.1%
Humerus	2	7.4%
Femur	8	29.6%
Leg	2	7.4%
Foot/ankle	5	18.5%
Polytraumatized	7	25.9%
Membrane infection (yes)	10	37.0%
**Location of infection**		
Senior Members	6	22.2%
Lower members	21	77.8%
Germs in culture (yes)	25	92.6%
Surgery (no)	2.2 ± 2.8	0.0 – 15.0
Use of Antibiotics (days)	35.0 ± 15.1	15.0 – 74.0
**Classes of antibiotics**		
Cephalosporins (yes)	15	55.6%
Glycopeptides (yes)	10	37.0%
Aminoglycosides (yes)	6	22.2%
Penicillins (yes)	12	44.4%
Quinolones (yes)	10	37.0%
Other (yes)	21	77.8%
**Outcome**		
Cure	20	74.1%
Recurrence	4	14.8%
Amputation	2	7.4%
Death	1	3.7%

The germs found in the cultures, notably for *S. aureus* (n = 8; 29.6%), and polymicrobial infections (n = 7; 25.9%), as the most common results. The average antibiotic duration was 35 days and more than half of the patients (63.0%) used three classes of antibiotics. About 75% of patients showed good results, that is, they were cured. Seven patients had poor results (recurrence, amputation or death).

Table 2 presents the variables associated with the patient's hospitalization time. The hospitalization time was longer for patients who had infection in the lower limbs. From a clinical point of view, the size of the observed effect was moderate. On the other hand, hospitalization time was not associated with the sex and age of the patient, the fracture region, the presence of germs in the culture, diaphyseal infection or even the number of classes of antibiotics used.

**Table 2:** Variables associated with patient hospitalization time (n = 27).

Explanatory variables	Time of internation		p-value	SE
	<= 39 days	>39 days		
**Sex**				
Female (n=8)	50.0%	50.0%	0.71	0.07
Male (n=19)	57.9%	42.1%		
Age	46.8 ± 20.2	53.2 ± 16.8	0.38	0.34
**Fracture region**				
Senior Members (n=5)	100.0%	0.0%	0.08	0.43
Lower Members (n=15)	46.7%	53.3%		
Polytraumatized (n=7)	42.9%	57.1%		
**Diaphyseal Infection**				
No (n=17)	52.9%	47.1%	0.72	0.07
Yes (n=10)	60.0%	40.0%		
**Infection site**				
Lower Members (n=21)	42.9%	57.1%	0.01*	0.48
Senior Members (n=6)	100.0%	0.0%		
**Germs in culture**				
Yes (n=25)	56.0%	44.0%	0.87	0.03
No (n=2)	50.0%	50.0%		
**Use of Classes of Antibiotics**				
<=2 (n=10)	70.0%	30.0%	0.25	0.22
3 or + (n=17)	47.1%	52.9%		

SE: size of the effect;

* represents values of p<0.05, by independent Student t test (quantitative variables) and by Qui-Quarter test (qualitative variables).

The median time of antibiotic use was 34 days. No statistically significant explanatory variables were found for the duration of antibiotic use. A moderate and statistically significant positive correlation was observed between the number of days of antibiotic use and the time of hospitalization (r = 0.64; p<0.001; n = 27), suggesting that the longer the time of hospitalization, the greater the use of antibiotics.

The patients who used antibiotics for more than 34 days compared to those who used for a shorter period were those who stayed on average 60.1 ± 21.2 days interned compared to 37.2 ± 29 days interned for the rest (p = 0.03). There were no statistically significant differences between patients who were readmitted and those that were not readmitted.

When analyzed patients with cure (n=20) compared to others (recurrence, amputation or death), it was observed that the treated patients used a higher amount of cephalosporins (93.3% vs. 6.7%; p = 0.01; V = 0.49) and penicillin (100.0% vs. 0.0%; p = 0.02; V = 0.53). From a practical point of view, the size of the effect of this difference was moderate for the use of cephalosporin and large for the use of penicillin. For the other variables, age, sex, hospitalization time, antibiotic use time, fracture region, infection site, diaphyseal infection, germs in the culture and number of surgeries, no statistically significant differences were observed between the groups (p>0.05). (Table 3)

**Table 3:** Variables associated with the clinical outcome of patients (n = 27).

Explanatory variables	Outcome		p-value	SE
	Healed (n = 20)	Recurrence, amputation or death (n=7)		
**Sex**				
Female (n=8)	62.5%	37.5%	0.63	0.17
Male (n=19)	78.9%	28.1%		
Age	46.7 ± 15.8	58.1 ± 24.7	0.17	0.
Internship (days)	43.7 ± 22.9	61.0 ± 37.2	0.16	
**Surgery**				
<=1 (n=15)	80.0%	20.0%	0.66	0.15
2 or + (n=12)	66.7%	33.3%		
**Fracture Region**				
Upper members (n=5)	60.0%	40.0%	0.60	0.19
Lower members (n=15)	73.3%	26.7%		
Polytraumatized (n=7)	85.7%	14.3%		
**Diaphyseal Infection**				
No (n=17)	70.6%	29.4%	0.68	0.10
Yes (n=10)	80.0%	20.0%		
**Infection site**				
Lower Members (n=21)	76.2%	23.8%	0.63	0.09
Senior Members (n=6)	66.7%	33.3%		
**Germs in culture**				
Yes (n=25)	72.0%	28.0%	0.97	0.17
No (n=2)	100.0%	0.0%		
Use of Antibiotics (days)	43.7 ± 22.9	61.0 ± 37.2	0.16	
**Antibiotics class(yes)**				
Cephalosporins (n=15)	93.3%	6.7%	0.01*	0.49
Glycopeptides (n=10)	70.0%	30.0%	0.71	0.07
Aminoglycosides (n=6)	83.3%	16.7%	0.56	0.11
Penicillins (n=12)	100.0%	0.0%	0.008*	0.53
Quinolones (n=10)	60.0%	40.0%	0.36	0.25
Other (n=21)	66.7%	33.3%	0.15	0.32

SE: size of the effect;

* represents values of p<0.05, by independent Student t test (quantitative variables) and by Qui-Quarter test (qualitative variables).

On the questionnaire (Annex I) offered to the nine orthopedists, all responded and the data of the content validity analysis are presented in Table 4. Regarding the relevance of the items, the instrument presented a content validity coefficient of 0.91, above the reference cutting point, with CVC variation of 0.83 to 1.00.

**Table 4:** Content validity coefficient for questionnaire items for defining the infection treatment flow at the fractures fixation surgical site.

No	Items	Relevance
1	Protocol diagnostic criteria	89%
2	Classification of infections with less than 4 weeks as acute and more than 4 weeks as chronic	89%
3	Imaging evaluation with radiography for all patients	89%
4	Surgical treatment	100%
5	Empirical intravenous antibiotic therapy with vancomycin and cefepime	94%
6	Follow-up time of 1 year	83%
	CVC total scale	91%

CVC: Content validity coefficient.

## DISCUSSION

The results of the study highlighted the characteristics of the sample with post-osteosynthesis infection (POI) in the appendicular skeleton and facilitated the development of an assistance protocol, with high agreement among respondents. This initiative aims to standardize the diagnosis and treatment of POI, which is impactful after the treatment of traumatized patients, in order to decrease, after its implantation, the indicators of hospital stay and the consequent economic impact on the individual and the institution. In the proper management of these patients, attention is needed to the infectious picture because it impacts on the time of hospitalization, morbidity, disability, increase in social security expenses and mortality.^12^

On the profile of patients with POI, it is noted that the sample of this study is compatible with the pattern observed in other developing and sub-developed countries, since the majority of patients correspond to adult males and the most common site of infection were the lower limbs (77.8%), mainly the femur (33.3%).^13^ *S. aureus* is the main agent found in the microbiology of POI, reporting 29.6% of cases.^4^ In the cohort prospectus of Kuehl et al., 229 patients with a diagnosis of osteosynthesis-associated infection (27.6%), identifying *S. aureus* as the most common pathogen in general (41.9%), while polymicrobial infections corresponded to 29.8%.^14^ In addition, a similar percentage of infections caused by polymicrobial flora was observed (27.6%), consistent with the literature.

The patients who showed a good result (curation) accounted for 74.1% of the total, a lower percentage than the Swiss cohort (88.3%).^14^ There was association between curation and use of two or more antibiotics in treatment, compared to other patients with poor outcome (recurrence, amputation or death), notably for cephalosporins and penicillins, which reinforces the value of combined therapy, with the goal of a broad spectrum treatment due to a high resistance rate of up to 30%.^15^ Regarding readmission, it is understood that it can be used as a marker of treatment quality, but in this cohort was not considered an unfavorable outcome, since the patients were not necessarily readmitted for the treatment of the infectious background, e.g.

A statistically significant correlation between the number of days of antibiotic use and hospitalization time was expected, as venous antibiotic therapy is the main cause of hospitalization in these cases. The median hospitalization time was 48 days, the median use of antibiotics was 34 days. This data reinforces the need to optimize POI treatment, as the cost of infected cases can increase by 90% compared to uninfected cases,^15^ and the costs associated with long-term hospitalization (admissions and readmissions) accounted for 62% of total POI costs in Tibia.^5^

The main aspects of the cohort result for the development of the POI assistance protocol were: criteria for confirmatory and suggestive diagnosis of the infection, classification as acute or chronic, image evaluation, surgical treatment, antibiotic therapy and total hospitalization time. Evolution time is an important aspect in the pathogenesis of POI, as the biofilm maturity occurs over weeks and determines the effectiveness of the antimicrobials; bone consolidation is crucial for the cure of infection and occurs over the course of weeks to months.^7^ For standardization purposes, the time of differentiation between acute and chronic infection of the protocol was established as four weeks, despite the controversy of this temporal classification criterion and the minimum follow-up time one year. Despite the divergence of these two outcomes in the literature,^6^ both achieved CVC of 89 and 83% respectively.

**Figure 2:**
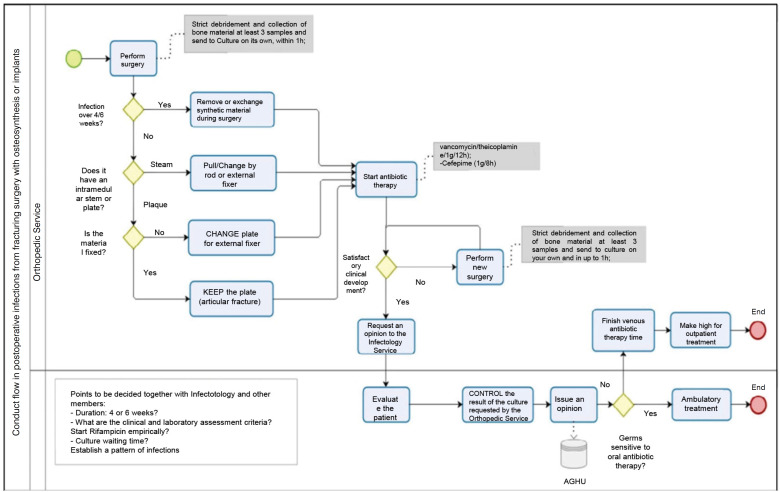
Fluxogram of post-osteosynthesis infections treatment protocol (POI).

As a limitation of the study, we highlight the small number of patients, differentiation between closed fractures of exposed patients, absence of classification of fractures and non-evaluation of clinical comorbidities that could influence the outcome (such as diabetes mellitus and peripheral vascular diseases).^14^ The main positive point was the development of a standardization to POI care. As future perspectives, the implementation of the protocol (Figure 2), resulting from the analysis of demographic results in accordance with the agreement of the assisting physicians, aims to decrease the average time of hospitalization, allowing for increased use of oral antibiotics replacing intravenous in some situations, with reduced days of hospitalization, and reduced costs.^16^ This protocol will be the subject of a prospective study to be started after its implementation.

## CONCLUSION

The characterization of post-osteosynthesis infection cases was similar to the literature and explained the items to be addressed in the development of the instrument. The high value of the content validity coefficient demonstrates high agreement with the proposed protocol, which was implemented in the institution.

## Annex 1 Question for defining the treatment flow of on-site infections.

### Fixing fractures surgical

**Confirmative criteria for post-osteosynthesis infections (1PO).**

1. Fistula, collection or rupture of the soft parts with exposure of the focus of the fracture or implant (medium communication with bone or implant).
2. Purulent drainage of the wound or the presence of pus during surgery.
3. Phenotypically similar pathogens, identified by culture from at least two separate bone/deep tissue fragments/implants (including sonication liquid). Samples taken during an operational intervention. In the case of fabric, at least 3 samples must be sent, in appropriate means, each with clean instruments, do not carry out superficial harvest or fistulous route. In cases of arthritis in a joint adjacent to a fractured bone, fluid samples obtained by sterile puncture can be included for study.
4. Presence of microorganisms in deep tissues captured during the surgical intervention, confirmed by histopathology examination using specific coloring techniques for bacteria or fungi.

**Suggestive 1PO criteria:**

1. Clinical signs: Pain (no weight support, increasing over time, new symptom), local redness, local swelling, local temperature increase, fever (single temperature measurement of 38.3 C).
2. Radiological signs: bone lyse (at the site of fracture, around the implant), loosening of the implant, seizure (occurring over time), failure to progress bone healing (non-union), presence of periosteal bone formation (e.g., in different locations from the site of fracture)
3. A pathogenic organism identified by culture from a single tissue/implant sample (including sonication fluid), always following the proposed standards for collection and evaluation.
4. High serum inflammatory markers: in musculoskeletal trauma conditions, these should be interpreted with caution. They are included as suggestive signs in case of secondary increase (after an initial decrease) or consistent increase over a period, and after exclusion of other infectious or inflammatory focuses: Erythrocyte sedimentation rate (HSS). White Blood Cell Count (WBC), C-Reactive Protein (CRP)
5. Persistent, increasing or recent drainage beyond the first days after surgery, without other explanation.
6. Recent onset of joint stroke in patients with fracture. Surgeons should be aware that ICF may present itself as an adjacent focus for infectious arthritis in the following cases: Implant material that penetrates the joint capsule (e.g., femoral implant) and intraarticular fractures.

I agree ()

I disagree ()

Why?_____________________________________________________________________________________________________________________________________________________________________________________________________________________________________

**Pathogenesis/ Classification**.^7-9^

The formation of biofilm on the surface of the implant or seizure material is crucial for the pathogenesis of infection in the presence of post-fractural implants.

The time for biofilm maturation is not yet fully established or follows a standard in the literature, so the classification of infections into acute (immature biofilm) and chronic infections (mature biofilm) is arbitrary in the treatment of POIs.

We established in our protocol that if the infection occurs within 4 weeks of surgery, we will characterize as acute and if it is more than 4 weeks, we will characterize as chronic.

I agree ()

I disagree ()

Why?_____________________________________________________________________________________________________________________________________________________________________________________________________________________________________

**Evaluation by Image**:^6^

The imaging examinations, performing the conventional radiography in the routine and at the beginning of treatment, already the tomography, only in selected cases, for conducting our patients. We do not see practical applicability for the execution of magnetic resonance imaging.

I agree ()

I disagree ()

Why?_____________________________________________________________________________________________________________________________________________________________________________________________________________________________________

**Treatment:** ^3,10,11^

There is no treatment option for ICF without initial surgery, which aims to achieve the following: strict debridement as an essential step; collection of tissue samples for culture and histopathological examination, preferably taken directly from the bone at the fracture site; at least three samples should be collected; superficial swabs or fistulous tracts should never be evaluated.

We prioritize the time factor when deciding whether to keep, remove, or replace implant material, using 4 weeks as the cutoff point. An exception is made for intramedullary stems, which will be removed or replaced at any time due to the difficulty of debriding the medullary canal.

The use of antibiotics on site will not be used in all cases, it will be used to fill bone losses and will be done with cement with antibiotics.

I agree ()

I disagree ()

Why?_____________________________________________________________________________________________________________________________________________________________________________________________________________________________________

**Antibiotic therapy**.^3,4,7,9^

After surgical debridement and obtaining tissue samples for culture, treatment should be initiated with empirical antibiotics.

As a rule, the initial empirical therapy should include an agent targeting gram-positive bacteria (such as a lipo- or glycopeptide) and an agent effective against gram-negative bacilli. The therapy should then be adapted according to the culture results as soon as possible, with ongoing support and monitoring by the infectious disease team.

Maintained intravenously for at least 4 weeks, followed by oral administration, where possible, and outpatient treatment, up to a total of 6 to 12 weeks, depending on the maintenance of the implant.

I agree ()

I disagree ()

Why?_____________________________________________________________________________________________________________________________________________________________________________________________________________________________________

**Ambulatory follow-up (follow-up):**

A year.

I agree ()

I disagree ()

Why?_____________________________________________________________________________________________________________________________________________________________________________________________________________________________________
